# Real-World Evaluation of the Safety and Effectiveness of 2.3% Hypertonic Saline Soft Mist Spray for Sino-Nasal Symptoms

**DOI:** 10.7759/cureus.55302

**Published:** 2024-03-01

**Authors:** Dipak Gandhi, Alok Semwal, Vikas Agrawal, Ravindra Jain, Harsh Srivastava, Preeth Shetty, Ravindra Chopra, Ravi Mehta, Chaitali Pilliwar, Ashok Jaiswal

**Affiliations:** 1 Pediatrics, Atharv Child Care, Mumbai, IND; 2 Pediatrics, Semwal Child Clinic, Dehradun, IND; 3 Pediatrics, Cradle Clinic, Meerut, IND; 4 Pediatrics, SR Hospital, Muzaffarnagar, IND; 5 Pediatrics, Saroj Medical Institute (SMI), Delhi, IND; 6 Pediatrics, Mallige Child Care Center, Bangalore, IND; 7 Pediatrics, Muskan Children Hospital, Surat, IND; 8 Medical Affairs, Zydus Healthcare Limited, Mumbai, IND

**Keywords:** allergic rhinitis, hypertonic saline, intra-nasal saline, sea salt saline, tnss

## Abstract

Introduction and aims

Mildly hypertonic saline is more effective in relieving symptoms of nasal congestion compared to placebo or isotonic saline. Recently, a unique device, delivering a soft mist of 2.3% hypertonic sea-salt saline (Nasoclear PureHale^TM^; Zydus Healthcare Ltd., India) has been introduced in India. The device uses a power-less manual technique to release the saline as a soft mist at 1 ml/min.

Methods

This is a retrospective, multi-centric, single-arm study to evaluate the safety and effectiveness of 2.3% hypertonic sea-salt saline nasal irrigation delivered through a soft mist device in patients with sino-nasal symptoms. This is an analysis of data of 130 patients collected from the medical records of 11 practicing pediatricians across India.

Results

The mean age of the patients was 5.23 ± 4.24 years; 63 % were boys and 37% were girls (n = 130). The mean reduction in total nasal symptom score (TNSS) at follow-up from baseline was 6.28 ± 0.18 (median days = 7) (95% CI = 5.92 to 6.64; p<0.0001; mean TNSS at baseline = 7.75 ± 2.01, mean TNSS at follow-up = 1.47± 1.30). Out of 130 patients, 33 patients (25.3%) showed complete improvement in TNSS, 93 patients (71.5%) had ≥ 50% improvement in TNSS while 4 patients (3.07%) showed <50% improvement in TNSS. The effectiveness of the device was rated as excellent (75%-100% improvement) and very good (50%-75% improvement) in 41 and 74 patients, respectively. It was rated as very easy to use and easy to use by 62 patients and 57 patients, respectively. One hundred nineteen patients (91.5%) were compliant with the prescribed frequency of the device and 110 patients (84.6%) were compliant with the prescribed duration of use of the device. No serious adverse events were reported; two patients reported mild side effects - stinging and irritation of the throat.

Conclusions

The 2.3% hypertonic sea-salt saline nasal irrigation delivered through the soft mist device was found to be well-tolerated and effective in patients with sino-nasal symptoms in real-world clinical settings.

Clinical trial number

The clinical trial number of this study is CTRI/2022/07/043751.

## Introduction

Allergic rhinitis (AR) is defined as Type 1 hypersensitive inflammation of the nasal mucosa, induced by exposure to allergenic substances, with at least two of the four cardinal nasal symptoms, e.g., sneezing, rhinorrhea, nasal itching, and nasal block, present for >1 hour per day on most or many days in a year [1]. As per Allergic Rhinitis and Its Impact on Asthma (ARIA) guidelines, allergic rhinitis can be of four types, based on symptom duration and severity: a) mild intermittent, b) mild persistent, c) moderate-to-severe intermittent, and d) moderate-to-severe persistent [2]. Different phenotypes of rhinitis have been identified based on etiology: allergic rhinitis, non-allergic non-infective rhinitis, infectious rhinitis, and idiopathic. Rhinitis has been found to be widely prevalent globally, including in India, and it affects patients across age groups. The reported incidence of allergic rhinitis in India ranges from 20% to 30% [3]. According to the Asia-Pacific Burden of Respiratory Diseases study, among 5250 adults, the majority of patients receiving care for respiratory disorders had allergic rhinitis (14%) followed by asthma (13.5%) and rhinosinusitis (5.4%) [4]. In the pediatric age group, an Indian study reported the prevalence of AR to be 11.3% in children aged 6-7 years and 24.4% in children aged 13-14 years. Rhinitis has been documented to affect the quality of life adversely in patients, with a substantial impact on the social, psychological, and economic aspects [5].

Pharmacologic treatment options for allergic rhinitis include oral and intranasal antihistamines, intranasal corticosteroids, leukotriene receptor antagonists, along with decongestants and oral corticosteroids in a particular group of patients [5]. Decongestants and intranasal cromolyn are not recommended for children and are used in adults for a short duration, limited to a few days. Second-generation antihistamines are preferred for the treatment of allergic rhinitis, as they effectively improve symptoms such as sneezing, itching, and rhinorrhea, are non-sedative, and have a better safety profile [6]. Although intranasal corticosteroids are commonly recommended, their peak effect may take several days to develop, and long-term use is associated with adverse effects on skeletal growth and adrenal activity, particularly in the pediatric age group.

Nasal saline irrigation is commonly recommended as a simple and inexpensive non-pharmacological therapy. It has been universally recommended for all ages in various guidelines. However, there is no clear differentiation in the use of nasal saline irrigation with respect to the severity of AR, i.e., mild, moderate, or severe. Intranasal use of saline alone or combined with traditional treatments for rhinitis and rhinosinusitis has been shown to improve symptoms and quality of life while decreasing the overall need for pharmacotherapy. It acts by softening and dislodging thick mucus and crusts, increasing the ciliary transport of mucus by enhancing ciliary beating frequency, and flushing out allergens, microorganisms, and inflammatory mediators [6]. Evidence suggests that seawater or mildly hypertonic saline is most effective in relieving symptoms of congestion compared to placebo or isotonic saline [7]. Although nasal saline use has been shown to be safe and effective, minor side effects, such as local irritation, itching, burning sensations in the nasal cavity, etc., have been reported. Most of these minor side effects can be ameliorated with modifications to the technique used for saline instillation and by adjusting the salinity [8]. In a meta-analysis of 4 trials involving 351 children with AR, a higher rate of adverse effects was reported in patients using 4.5% vs. 2.3% hypertonic saline [9].

In the present study, we aim to understand the safety and effectiveness of a unique power-less device that releases 2.3% sea-salt hypertonic saline in the form of a soft mist (Nasoclear PureHaleTM; Zydus Healthcare Ltd., India). The device can be used to release hypertonic saline at the rate of 1 ml/min in patients with sino-nasal symptoms.

## Materials and methods

Study design and participant enrollment

This was a retrospective, multi-centric, single-arm study, and the real-world data from clinical practice was retrospectively transcribed in the data collection form. Data from 130 patients from all age groups and genders, across multiple medical centers in India, was collected for analysis. As this study is a retrospective analysis of data collected as part of real-world clinical diagnosis and treatment, there was no consent form collected from the patients. Also, any personal data of the patient which can reveal the patient’s identification, was neither transcribed in the data collection nor used for analysis. Patients having indications for the application of hypertonic saline nasal sprays (Nasoclear PureHale^TM;^ Zydus Healthcare Ltd., India) with symptoms of nasal congestion at baseline, total nasal symptom score (TNSS) as ≥ 5 at baseline, and eligible to receive the prescribed nasal saline spray as per the physician’s discretion were included in the study. The indications included but were not limited to viral, bacterial, or allergic rhinitis and acute or chronic rhinosinusitis. No exclusion criteria were applied in this retrospective observational study. The study was approved by the Ethics Committee and was registered on the Clinical Trials Registry - India (CTRI/2022/07/043751).

Data collection

The baseline patient characteristics, including demographics and diagnosis details, were collected and transcribed. For efficacy assessment of 2.3% sea-salt hypertonic saline, at baseline, TNSS, co-medications, and prescribed frequency and duration of nasal spray were assessed. At a median follow-up of seven days (Days 5-10), changes in the values of these parameters in terms of change in mean TNSS, changes in mean number of co-prescribed medications, and reduction in the requirement of anti-histamines were recorded and assessed. Also, the Physician’s Global Assessment of Effectiveness was rated on a scale from 0 to 4, depending on the percentage of improvement of symptoms as per the physician. The effectiveness of the device was rated as excellent (score = 4) for 75%-100% improvement, very good (score = 3) for 50%-75% improvement, good (score = 2) for 25%-50% improvement, bad (score = 1) for <25% improvement, and very bad (score = 0) for no improvement [10]. To understand the compliance and ease of use of the device, the frequency and duration for which the device was used by the patient and the ease of using the device were assessed. This data was recorded by the treating physician after discussing it with the patient.

Statistical analysis

As this is a retrospective, real-world evaluation of the safety and effectiveness of a marketed device, there is no primary outcome measure nor any formal sample size calculation. GraphPad software (https://www.graphstats.net/) was used to analyze the data. Descriptive statistics, such as means, SD, and proportions, were performed. A paired t-test was used to calculate the change in parameters between baseline and follow-up such as change in mean TNSS and change in mean number of anti-histamine drugs prescribed.

## Results

Clinical characteristics and demographic data

One hundred thirty patients (N = 130) with sino-nasal symptoms were included in the study. The indications included but were not limited to viral, bacterial, or allergic rhinitis and acute or chronic rhinosinusitis. The mean age of the patients was 5.23 ± 4.24 years and the number of boys and girls was 82 (63%) and 48 (37%), respectively. The demographic details of the patients are represented in Table 1.

**Table 1:** Demographic details of patients URTI - Upper Respiratory Tract Infection; AR - Allergic Rhinitis; LRTI - Lower Respiratory Tract Infection; TNSS - Total Nasal Symptom Score; SD - Standard Deviation

Characteristics	Values
Total, N = 130	
Age (Years), Mean ± SD	5.23±4.24
Gender	
Male (%)	82
Female (%)	48
Diagnosis (n)	
URTI	62
AR	35
URTI + AR	5
LRTI + AR	6
Others	22
Co-medications (n)	
Antihistamine	61
Bronchodilator	37
Antibiotic	26
Antipyretic	16
Decongestant	6
Antiemetic	2
Antitussive	1
No co-medications	26
TNSS, Mean ± SD	7.75± 2.01

The TNSS of the patients was recorded at baseline and follow-up. The mean TNSS at follow-up was reduced to 1.47± 1.30 from the baseline value of 7.75 ± 2.01 (Median follow-up of 7 days; Mean Difference: 6.28 ± 0.18; 95% CI = 5.92 to 6.64; p<0.0001).

**Figure 1:**
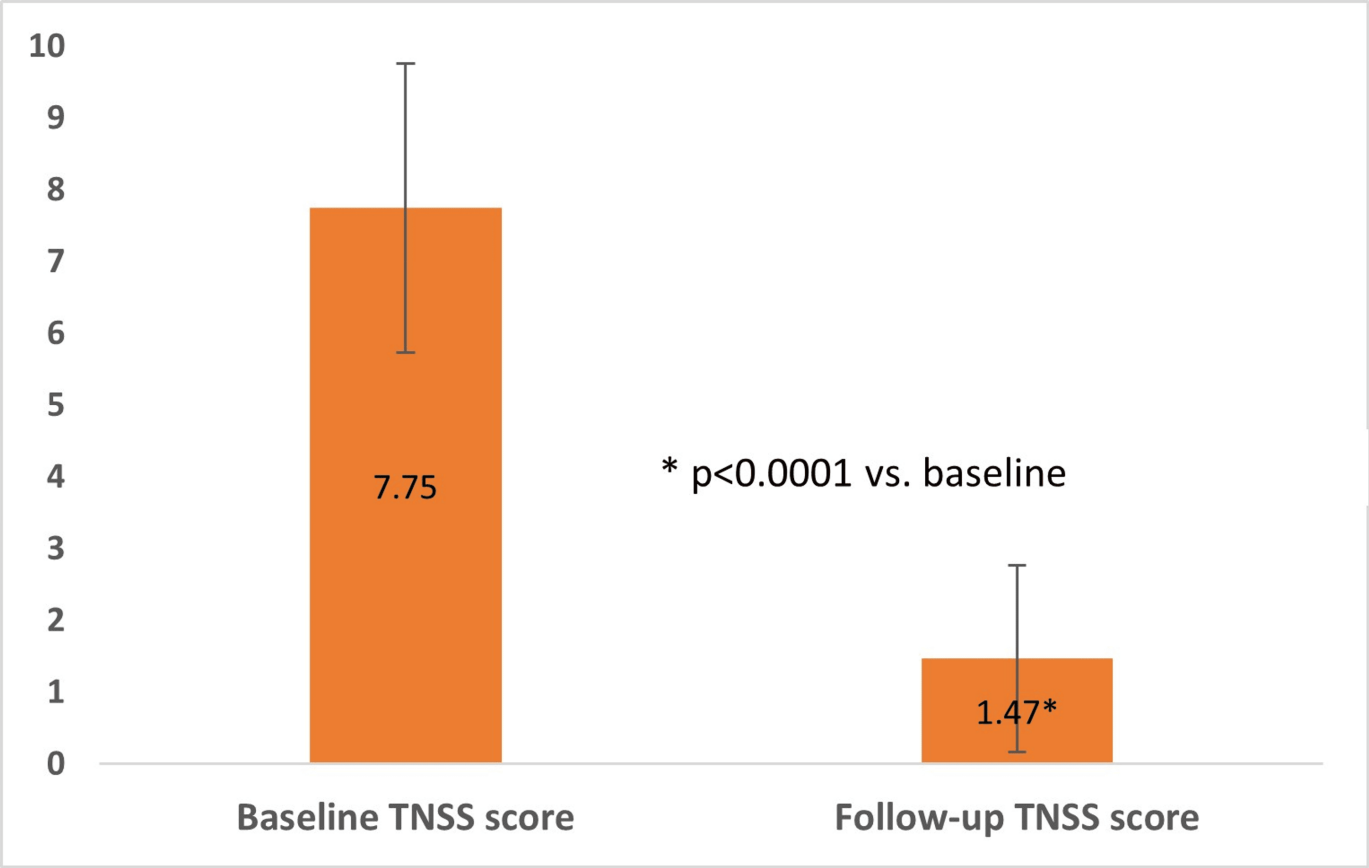
Change in mean TNSS from baseline to a follow-up of median seven days A paired t-test was used to calculate the change in mean TNSS (liner TNSS) from baseline to a follow-up of median seven days. TNSS - Total Nasal Symptom Score

**Figure 2:**
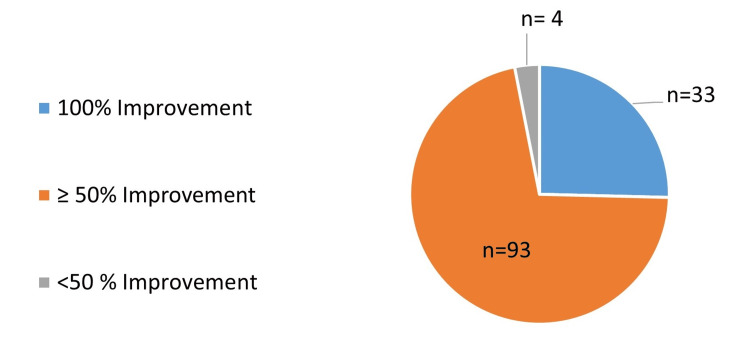
Percentage improvement in TNSS from baseline to a follow-up of median seven days TNSS - Total Nasal Symptom Score

The change in mean TNSS at follow-up is represented in Figure 1. Out of 130 patients, 33 patients (25.3%) showed complete improvement in TNSS, 93 patients (71.5%) had ≥ 50% improvement in TNSS while 4 patients (3.07%) showed <50% improvement in TNSS. The percentage improvement in TNSS at follow-up is represented in Figure 2.

**Figure 3:**
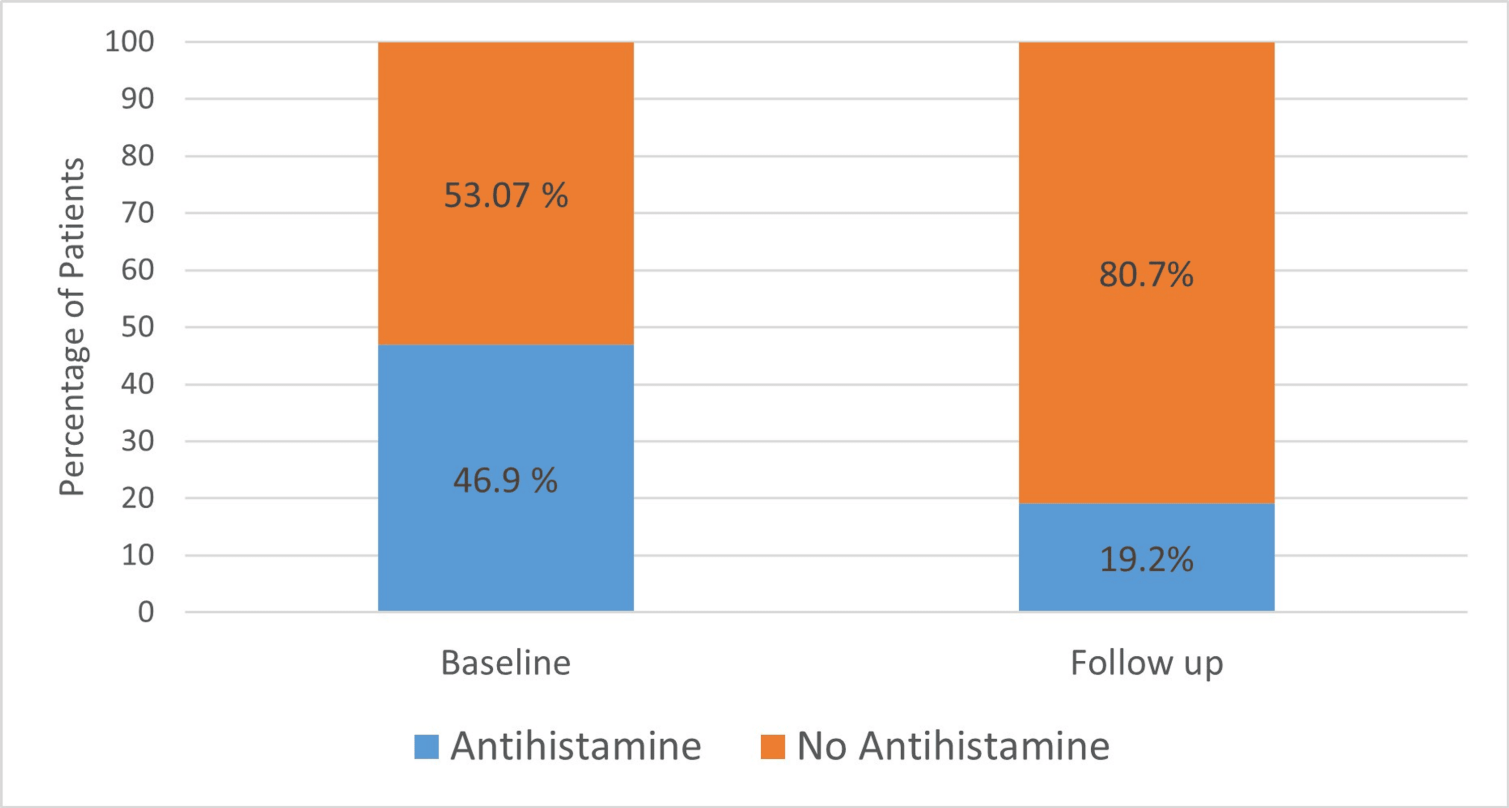
Percentage of patients receiving antihistamine at baseline and a median follow-up of seven days

At baseline, 80% of patients (104/130) were prescribed co-medications while only 48.4% (63/130 patients) were prescribed co-medications at follow-up, suggesting a 39% reduction in the use of co-medications with the use of adjuvant 2.3% hypertonic saline. The number of patients using anti-histamines also reduced at follow-up by 59%. Figure 3 shows the percentage of patients with a reduction in the requirement of anti-histamines at follow-up (median days of follow-up = 7) from baseline. At baseline, 46.9% of patients (n = 61 patients) received anti-histamines while at follow-up (median days of follow-up = 7), 19.2% of patients (n = 25 patients) were on anti-histamines.

**Figure 4:**
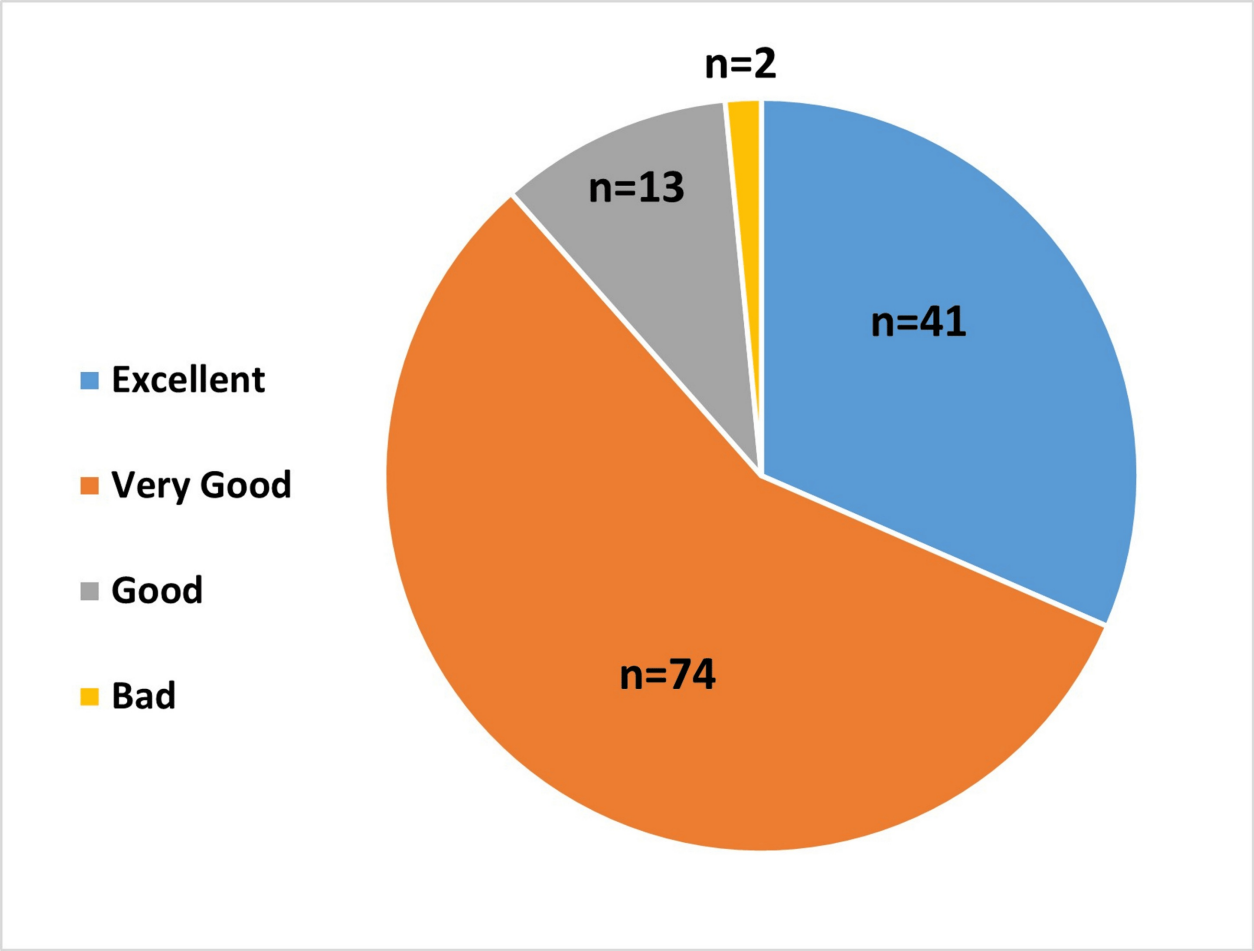
Physician’s Global Assessment of Effectiveness of the treatment via symptom improvement Excellent (75%-100% improvement), Very good (50%-75% improvement), Good (25%-50% improvement), Bad (0%-25% improvement)

The Physician’s Global Assessment of Effectiveness was rated on a scale from 0 to 4, depending on the percentage of improvement of symptoms as per the physician. The effectiveness of the device was rated as excellent (75%-100% improvement) and very good (50%-75% improvement) in 41 and 74 patients, respectively (Figure 4).

The ease of using the device was rated on a scale from 0 (very difficult to use) to 4 (very easy to use) depending on the feedback of the patient. It was rated as very easy to use and easy to use by 62 patients and 57 patients, respectively, while 5 patients had a neutral opinion and 6 patients found the device difficult to use.

Out of 130 patients, 119 patients (91.5%) were compliant with the prescribed frequency of the device and 110 patients (84.6%) were compliant with the prescribed duration of use of the device. No serious adverse events were reported; two patients reported mild side effects - stinging and irritation of the throat.

## Discussion

Nasal saline washes disrupt the viscous surface layer and remove mucus along with the particulate material embedded in it. The addition of saline to nasal solutions increases hydration in the deeper aqueous layer, boosting the underlying ciliary beat frequency and decreasing local inflammatory mediators. Previous investigations showed that hypertonic solution is more potent than isotonic saline in improving sinonasal symptoms, especially nasal congestion, rhinorrhea, cough, headache, and waking up during the night [11,12].

The 2.3% hypertonic saline significantly reduced TNSS at follow-up from baseline (mean reduction in TNSS = 6.28 ± 0.18, (95% CI = 5.92 to 6.64; p<0.0001). The findings were similar to a study that demonstrated hypertonic saline nasal irrigation (HSNI) improved patients’ nasal symptom scores (mean difference, 1.82 points after treatment; 95% CI, 0.35-3.30; I2 = 64%; p = 0.02) and showed better nasal symptom scores in the HSNI group compared to the isotonic group (mean difference, 1.22 points; 95% CI, 1.01-1.44; I2 = 0%; p < 0.001) [9]. In a randomized controlled trial performed as a pilot study, the TNSS scores in AR patients using hypertonic saline significantly decreased at week 2 and week 4 (p< 0.001; TNSS at baseline = 7.40 + 2.22; TNSS at week 2 = 1.83 + 0.44; TNSS at week 4 = 0.77 + 91) following initiation of nasal saline irrigation. This pilot study concluded that regular use of hypertonic nasal saline in AR patients for four weeks is safe and has superior efficacy to 0.9% isotonic nasal saline for alleviating congestion and improving quality of life scores [13]. The 2.3% hypertonic saline relieved sinonasal symptoms by 50% in 3.27 ± 1.07 days, which shows the efficacy of HSNI. The 2.3% HSNI showed complete (100%) improvement in TNSS in 33 patients (25.3%) and ≥ 50% improvement in 93 patients (71.5%).

In our study, 46.2% of patients were prescribed anti-histamines at baseline; at the end of a median follow-up of 7 days, 19.2% of patients were prescribed anti-histamines. A systematic review and meta-analysis showed a significant reduction in anti-histamine use in patients using HSNI (RR, 0.68; p = 0.02) as compared to those not using it, although the benefits were not different between the hypertonic and isotonic saline treatment groups. Only two patients in our study had mild side effects such as stinging sensations and irritation of the throat. Adverse effects are a major concern when treating children; however, the adverse effects (nasal irritation, burning sensation, or nose bleeding) reported in the systematic review did not vary between patients treated with isotonic and hypertonic nasal saline. Thus, in terms of the alleviation of nasal symptoms and adverse effects, hypertonic saline was superior to isotonic saline in treating children with allergic rhinitis [9].

In our study, we focused on patients receiving nasal saline as adjuvant therapy along with other medications as indicated, and all patients received nasal saline. A systematic review revealed a statistically significant difference in treatment outcomes between the administration of saline in conjunction with medication compared to medication alone among adult patients. Specifically, the standardized mean difference (SMD) was calculated to be -1.30, with a 95% confidence interval ranging from -1.89 to -0.71. This indicates that the therapeutic efficacy of saline combined with medication was notably superior to that observed with medication alone. However, in the children group, the difference in symptom scores was not statistically significant between the two groups [14]. However, in our study, the patients (mean age: 5.23 ± 4.24 years) who received saline + medication therapy (n = 104) showed a significant reduction in symptom score (TNSS) at follow-up (p< 0.0001).

Multimodality treatments are required in treating AR, and saline irrigation is regarded as second-line adjuvant therapy for patients with inadequately controlled symptoms. Nasal saline is beneficial in treating rhinitis, acute and chronic sinusitis, and other upper respiratory tract illnesses brought on by inflammatory mediators [15]. The mechanical stimulus involved in the spray application of saltwater plays a role in achieving the beneficial effect by causing neuronal changes in the immunologic process [16]. While the optimal concentration of saline used for irrigation is unknown, our study demonstrated the effectiveness of 2.3% hypertonic saline nasal irrigation. Our study had some limitations. It was limited by its retrospective nature, lack of comparability, and absence of comparison between different saline concentrations. Additionally, the small sample size constrained the generalizability of our findings. Nonetheless, our study provides valuable insights and sets the stage for future prospective research to further explore the potential benefits of saline therapy in this context.

## Conclusions

The 2.3% hypertonic sea-salt saline spray delivered through the soft mist device improved patients’ symptoms in terms of TNSS reduction in patients with sino-nasal symptoms. The hypertonic saline spray also showed a reduction in the use of anti-histamines in these patients. It was well tolerated with no major adverse effects. Based on our findings, 2.3% hypertonic nasal saline spray can be a reasonable adjunctive treatment for patients with allergic rhinitis and patients having sino-nasal symptoms.
