# Correction: Real-World Evaluation of the Safety and Effectiveness of 2.3% Hypertonic Saline Soft Mist Spray for Sino-Nasal Symptoms

**DOI:** 10.7759/cureus.c239

**Published:** 2025-08-13

**Authors:** Dipak Gandhi, Alok Semwal, Vikas Agrawal, Ravindra Jain, Harsh Srivastava, Preeth Shetty, Ravindra Chopra, Ravi Mehta, Chaitali Pilliwar, Ashok Jaiswal

**Affiliations:** 1 Pediatrics, Atharv Child Care, Mumbai, IND; 2 Pediatrics, Semwal Child Clinic, Dehradun, IND; 3 Pediatrics, Cradle Clinic, Meerut, IND; 4 Pediatrics, SR Hospital, Muzaffarnagar, IND; 5 Pediatrics, Saroj Medical Institute (SMI), Delhi, IND; 6 Pediatrics, Mallige Child Care Center, Bangalore, IND; 7 Pediatrics, Muskan Children Hospital, Surat, IND; 8 Medical Affairs, Zydus Healthcare Limited, Mumbai, IND

The Ethics Statements and Conflict of Interest Disclosures has been corrected as follows. The Payments section now includes the following disclosure: This study was funded by Zydus Healthcare Ltd., India. This was previously disclosed in the Financial Relationships section, and has been added to the Payments section as well to avoid potential confusion.

